# Evaluation of the Freestyle Optium Neo H point‐of‐care device for measuring blood glucose concentrations in sick calves

**DOI:** 10.1111/jvim.15794

**Published:** 2020-05-18

**Authors:** Tolga Karapinar, Kenan Cagri Tumer, Sébastien Buczinski

**Affiliations:** ^1^ Department of Internal Medicine, Faculty of Veterinary Medicine Firat University Elazig Turkey; ^2^ Département des sciences cliniques, Faculté de médecine vétérinaire Université de Montréal Saint‐Hyacinthe Québec Canada

**Keywords:** accuracy, bovine, glucometer, precision, sensitivity, specificity

## Abstract

**Background:**

Data on the performance of a glucometer in calves with different diseases are currently lacking.

**Objective:**

The primary objective of this study was to evaluate the reliability of a point of care glucometer in calves affected by different diseases relative to a traditional bench‐top autoanalyzer.

**Animals:**

One hundred ninety‐six calves with different disorders in a referral hospital.

**Methods:**

Prospective study. Venous blood samples were used for the determination of glucose concentrations in blood and plasma using the Freestyle Optium Neo H and autoanalyzer, respectively. Data were subjected to Passing‐Bablok regression and Bland‐Altman plots. The Freestyle Optium Neo H was the test method and the autoanalyzer was the reference method. The diagnostic performance of the glucometer relative to the autoanalyzer was assessed using 3 different plasma glucose concentrations.

**Results:**

The Passing‐Bablok regression for the glucometer against the reference method revealed the presence of both proportional bias (1.12; 95% confidence interval [CI], 1.07‐1.18) and constant bias (−11.25; 95% CI, −16.0 to −7.70). The glucometer yielded 92.2%‐100% sensitivity and 86.4%‐96% specificity for the assessing glucose concentration based on different concentration thresholds.

**Conclusions and Clinical Importance:**

The Freestyle Optium Neo H showed proportional and constant biases relative to the reference method. The glucometer showed poor performance according to criteria recommended by the International Standards Organization and the American Society for Veterinary Clinical Pathology. However, the glucometer determined hypoglycemia with high sensitivity and specificity therefore it might be used to diagnose hypoglycemia in calves with different diseases until calf‐specific POC glucometers are developed.

**Abbreviations**
AUC: area under the receiver operating curve
CV: coefficient of variation
Hct: hematocrit
POC: point‐of‐care
ROC: receiver operating characteristics
Se: sensitivity
Sp: specificity
TE: obs total observed error
TP: total protein

## 1 INTRODUCTION

Commercially available portable blood glucose meters are small, convenient, and cost effective devices to measure blood glucose concentrations. Glucometers can yield rapid results and can potentially assist clinicians to make short‐term therapeutic strategies especially in hypoglycemia. Therefore, point‐of‐care(POC) glucometers may be used in ambulatory clinics and farm settings in bovine medicine. Most commercially available glucometers are designed for human use. In spite of the fact that glucometers employ conversion algorithms to report the result as a plasma value, they measure the glucose concentrations in whole blood. These algorithms used by glucometers are based on intraerythrocyte to plasma glucose ratio that varies with species and age.^1^, ^2^ Evaluation of different glucometers designed for human beings was made in horses, foals, sheep, and goats.^3^, ^4^, ^5^, ^6^ The accuracy of blood glucose concentrations from different glucometers was also assessed in dairy cows.^7^, ^8^, ^9^ However, difference intraerythrocyte to plasma glucose ratio between adult cattle and calves might cause different glucometers' performance in calves.^1^, ^9^

Moreover, hematocrit (Hct) might affect the accuracy of glucometers. In human blood samples, increased Hct can cause decreases in blood glucose measurements and vice versa.^10^, ^11^ Changes in Hct seen in calves with different diseases such as diarrhea, and infection might therefore affect agreement of glucometer relative to reference method. Data on the performance of a glucometer in calves with different diseases are currently lacking.

Calves may have wide variation of their glycemia with diseases. Hypoglycemia is particularly common in acute conditions in neonates and is significantly associated with nonsurvival in ill neonatal calves.^12^ Therefore, it should be accurately recognized during the clinical examinations and urgently treated by the clinicians because of life‐threatening consequences. Hypoglycemia cannot be diagnosed based on only clinical signs and detection of hypoglycemia requires blood glucose measurements. It was nearly impossible to measure plasma glucose concentrations in ambulatory clinics and field settings until recent years because the use of special laboratory equipment is necessary. Improvements in POC technology provide veterinarians measurement of blood glucose concentrations in field conditions. Because POC glucometers can potentially give rapid results, POC glucometers which can accurately identify hypoglycemia in sick calves might be extensively used by the veterinarians in ambulatory clinics and farm settings. The Freestyle Optium Neo H (Abbott Diabetes Care Inc, Alameda, California) is a handheld glucometer designed for people to quantitatively measure glucose in whole blood samples and yields a rapid result within 5 seconds.

The objectives of this study were to (a) evaluate the agreement of the point of care Freestyle Optium Neo H in calves affected by different diseases relative to a traditional bench‐top autoanalyzer (Cobas C501 autoanalyzer, Roche, Mannheim, Germany) and (b) determine sensitivity (Se) and specificity (Sp) of the Freestyle Optium Neo H relative to Cobas C501 using 3 plasma glucose concentrations for the hypoglycemia classification based on previously determined thresholds.^13^, ^14^, ^15^, ^16^

## 2 MATERIALS AND METHODS

### 2.1 Animals

One hundred ninety‐six calves (<1 month age) with different diseases referred to Firat University Teaching and Training Animal Hospital between January 2018 and April 2019 were used in this prospective study. Calves with different diseases and different degrees of dehydration were selected to obtain wide range of blood glucose concentrations, Hct values, and plasma total protein (TP) concentrations. Calves that previously received glucose solutions before taking the blood samples and calves with blood glucose concentration ≤20 mg/dL measured by the glucometer were excluded from the study. This experiment was approved by the Firat University Ethics Committee on Animal Experimentation.

### 2.2 Blood samples

Blood samples were taken from the jugular vein of all calves before starting the treatment. A portion of the blood sample was transferred to test tubes including lithium heparin for plasma separation. The glucometer readings at the calf‐side were conducted on whole blood with no preservative immediately after the blood collection. After centrifugation of the test tubes at 3000*g* for 10 minutes, the plasma samples were stored at −20°C for plasma glucose and plasma TP concentration measurements within 7 days using an autoanalyzer. The interval from blood collection until plasma separation in the laboratory was maximum 20 minutes for all samples.

### 2.3 Blood glucose measurements by the glucometer

Blood glucose measurements were performed on blood samples without preservatives by the Freestyle Optium Neo H. Single‐use reagent strips were used for the Freestyle Optium Neo H. A drop of blood samples was placed on the test strip. All blood glucose measurements were made with 1 POC glucometer. The measurement range of the glucometer was 20 to 500 mg/dL. When blood glucose concentration was too low to be read by the glucometer, the glucometer gave LO message. Glucose Hct range was 15 to 65% according to manufacturer's manual for the glucometer. The glucometer displayed glucose in mg/dL in 5 seconds. The Freestyle Optium Neo H uses glucose amperometric technology to quantitatively measure the glucose concentration in whole blood samples. Briefly, the glucose biosensor recognizes the glucose present in blood samples by means of the glucose spesificity of the enzyme glucose dehydrogenase present on the glucose test strip. The electrons liberated by this reaction are transferred to the glucometer where they are read as a small electrical current. The size of the current is directly proportional to the concentration of the glucose in the applied blood sample. When blood sample applied to test strip is too small the glucometer gives error message.

The intra‐assay coefficient of variation (CV) of Freestyle Optium Neo H was determined from 7 replicates of low (mean, 38 mg/dL) and moderate (mean, 116 mg/dL) blood glucose concentrations.

### 2.4 Measurement of plasma glucose concentration, Hct, and plasma protein

#### 2.4.1 Concentration

Plasma glucose concentrations were measured by a traditional bench‐top autoanalyzer (Cobas C501). The Cobas C501 utilizes hexokinase enzyme and photometric method principles. The analytical measurement range of the autoanalyzer was 2 to 750 mg/dL. The intra‐assay CV of the Cobas C501 was also calculated from 7 replicates of low (mean, 24 mg/dL) and moderate (mean, 117 mg/dL) blood glucose concentrations.

Hematocrit measurement was performed once using capillary microhematocrit tubes after centrifugation for 5 minutes. Plasma TP concentrations were determined by the Cobas C501. The Cobas C501 uses biuret method and colorimetric assay. The analytical measurement range of the autoanalyzer was 0.2 to 12 g/dL. Because it has been previously established that intracorpuscular to plasma glucose ratio is higher in calves younger than 1 week of age,^1^, ^2^ we further identify the calves as <1 week old or 7 days of age or more because we anticipate that this covariate could potentially impact the relationship between Freestyle Optium Neo H and autoanalyzer.

### 2.5 Statistical analysis

Normality of the data was assessed with the Shapiro‐Wilk test. Normally distributed data were expressed as mean ± SD and nonnormally distributed data were expressed as median and range. The analyses were reported in mean ± SD(TP and Hct) or median (range) for blood, plasma glucose concentrations, and total observed error (TEobs) accordingly. The autoanalyzer was considered the reference method, whereas the Freestyle Optium Neo H served as the test method. Passing‐Bablok regression analysis was performed to evaluate the relationship between blood glucose concentrations and plasma glucose concentrations.^17^ Cumulative sum test (Cusum test) for linearity was also assessed with the Passing‐Bablok regression analysis. The agreement between the glucometer and the reference method was evaluated by Bland‐Altman plots.^18^ Receiver operating characteristic (ROC) curves were developed at the different cutoffs plasma glucose concentration of <72,^16^<58,^14^ and <40 mg/dL^13^, ^15^ (hypoglycemia) to determine Se and Sp of the Freestyle Optium Neo H at the highest Youden index, which minimizes the number of misclassificatied samples.^19^ The Youden index was calculated as Se + Sp‐1. The area under the curve (AUC) for ROC curves was calculated to measure diagnostic test performance of the Freestyle Optium Neo H. AUC > 0.90 was considered highly accurate, AUC between 0.70 and 0.90 was considered moderate accurate, AUC between 0.5 and 0.7 was considered low accurate, and AUC ≤ 0.5 was considered a chance result.^20^

In order to explore the potential impact of the glucose concentration as measured by the reference method, Hct and plasma TP concentration on the dependent variable (blood glucose concentration measured by the Freestyle Optium Neo H) a multivariable linear regression was performed. The difference percentage (DGlu (%) = (GluPOC‐GluRef)/GluRef)) was determined as the dependent variable. The initial model that was tested was:

DGlu=HCT+TP+Age+TP×HCT

The interaction between TP and Hct was a priori put in the model for accounting on these parameter changes in many different calves' diseases.^9^ The age (dichotomous <7j, versus ≥7j was also considered as a potential covariate. The regression modeling was performed using lme4 package from the R statistical software.^21^ The covariates were removed sequentially until the final model having all remaining variables with *P*‐value <.05. Distribution of the residuals was visually checked for normality. The fit of the model was determined using adjusted R‐squared.

The glucometer performance was also analyzed in compliance with the 2013 standard (ISO 15197‐2013) from the International Standards Organization in which 95% of the individual glucose results for the glucometer should fall within 15 mg/dL of the reference measurement for glucose concentrations when plasma glucose concentration <100 mg/dL and within 15% of the reference measurement for glucose concentrations when plasma glucose concentration >100 mg/dL.^22^ The performance of the Freestyle Optium Neo H was also assessed using a calculation of TEobs as follows: TEobs = 2 × intra‐assay CV (%) + bias (%).^23^ The bias was calculated according to the formula: bias% = [(reference method − glucometer)/reference method] × 100. TEobs% should be ≤20% in all glucose measurements.^23^, ^24^

Statistical analyses of data were performed using MedCalc (MedCalc Statistical Software version 19.03, MedCalc, Ostend, Belgium) and R software (R Core Team [2017]; R: A language and environment for statistical computing; R Foundation for Statistical Computing, Vienna, Austria, https://www.Rproject.org/). The level of *P* < .05 was considered significant.

## 3 RESULTS

Tentative diagnoses were diarrhea (n = 177), infection (n = 15), prematurity (n = 2), congenital defect (n = 1), and ruminal drinking (n = 1). Three calves that previously received glucose solutions before collecting the blood samples and 13 calves that had blood glucose concentration below the detection limit of the glucometer (≤20 mg/dL) were excluded from the present study.

The intra‐assay CV of the Cobas C501 for the mean of 24 and 117 mg/dL plasma glucose concentration was 3.7 and 1.2%, respectively. The intra‐assay CV of the Freestyle Optium Neo H for the mean of 38 and 116 mg/dL blood glucose concentration was 3.0 and 3.9%, respectively.

Nonnormally distributed data were observed in plasma and blood glucose concentrations. Plasma glucose concentrations measured by the Cobas C501 ranged from 18 to 155 mg/dL (median, 85 mg/dL). Blood glucose concentrations measured by Freestyle Optium Neo H ranged from 22 to 169 mg/dL (median, 80 mg/dL). Hct ranged from 10 to 55% (mean ± SD, 33.6 ± 8.0%) and plasma TP concentrations ranged from 3.1 to 10.6 g/dL (mean ± SD, 6.2 ± 1.5 g/dL).

**FIGURE 1:**
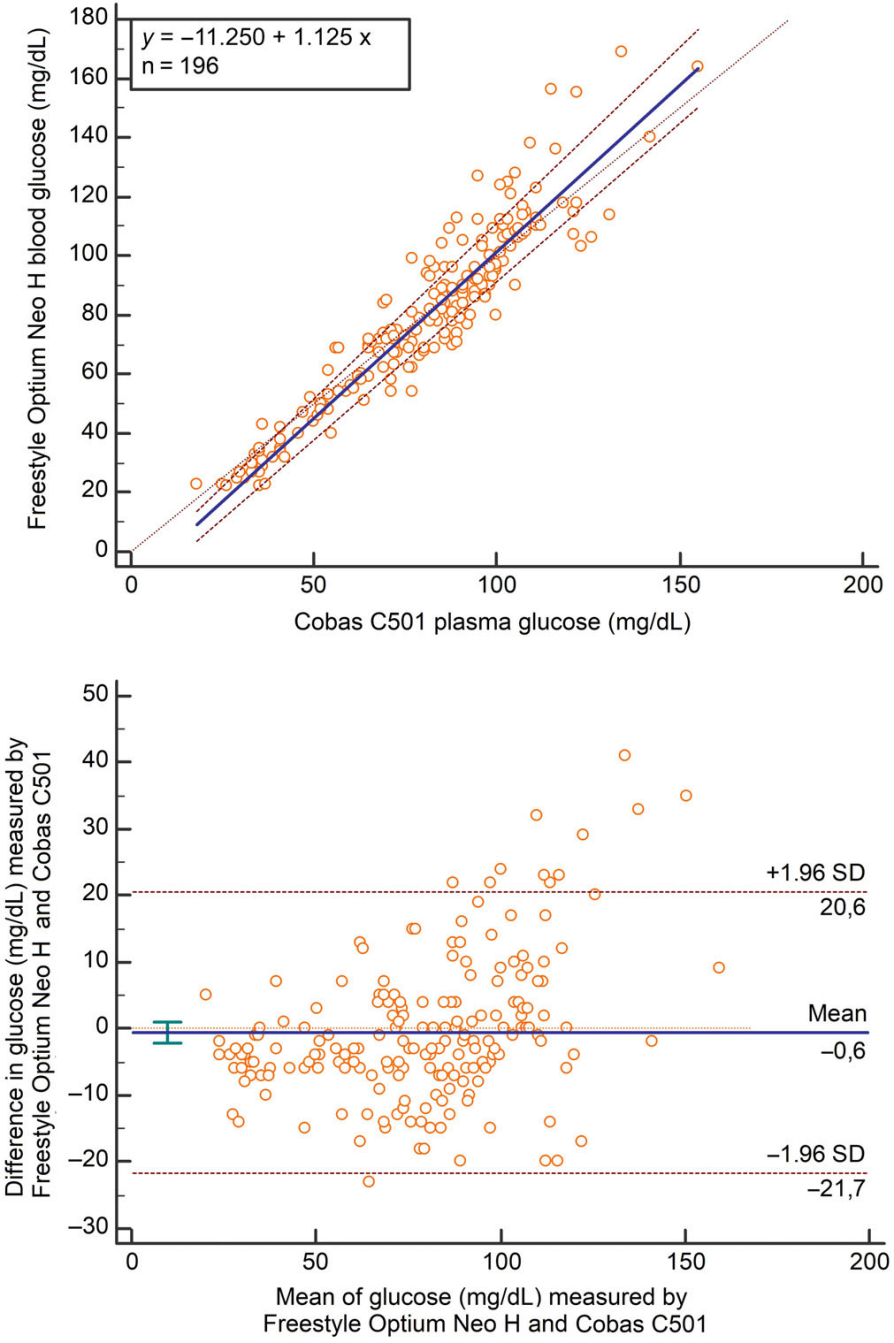
The Passing‐Bablok regression analysis (top) of the blood glucose concentrations determined using the POC glucometer versus plasma glucose concentrations measured using the autoanalyzer (n = 196). The diagonal dashed line is the identity line, the solid line represents the best fit, and the dashed lines represent the 95% confidence interval (CI). The Bland‐Altman plot analysis (bottom) of differences between blood glucose concentrations determined using the POC glucometer and plasma glucose concentrations determined using the autoanalyzer against their averages. The solid horizontal, straight line is the mean bias, the thick dashed line is the best fit line, the short horizontal lines represent the 95% CI of the bias, and the horizontal dashed lines represent the 95% CI of agreement

The Passing‐Bablok regression equation fitting plasma (X) and blood (Y) glucose concentration [95% CI] was *Y* = −11.25 [−16.0/−7.70] + 1.12 [1.07/1.18] × *X*(Figure 1). Residual SD (RSD) was 7.08 [−13.89/13.89]. The Bland‐Altman difference plot revealed that the mean bias was −0.6 mg/dL (95% CI, −2.09 to 0.95 mg/dL), but visual inspection of the graph revealed the presence of positive proportional bias (Figure 1). The 95% limits of agreement were 20.6 to −21.7 mg/dL.

Table 1 summarizes Se and Sp of the Freestyle Optium Neo H for hypoglycemia and additional test characteristics (AUC, Youden index).

**TABLE 1:** Receiver operating characteristic analysis for the Freestyle Optium Neo H in sick calves (samples, n = 196)

Threshold glucose concentration^a^ (n = 196)	Optimized Cutoff^b^ (mg/dL)	Sensitivity (95% CI)	Specificity (95% CI)	AUC^c^ (95% CI)	*P*	J^d^
40 mg/dL (n = 25)	≤43	100 (83.9‐100)	96 (91.9‐98.4)	0.997 (0.975‐1)	<.0001	0.96
58 mg/dL (n = 45)	≤54	92.7 (80.1‐98.5)	97.4 (93.5‐99.3)	0.991 (0.965‐0.999)	<.0001	0.90
72 mg/dL (n = 68)	≤72	92.2 (82.7‐97.4)	86.4 (79.3‐91.7)	0.966 (0.930‐0.987)	<.0001	0.79

a Plasma glucose concentration determined by the reference method.

b Optimized cutoff value for the Freestyle Optium Neo H.

c Area under the ROC curve.

d Youden index.

**FIGURE 2:**
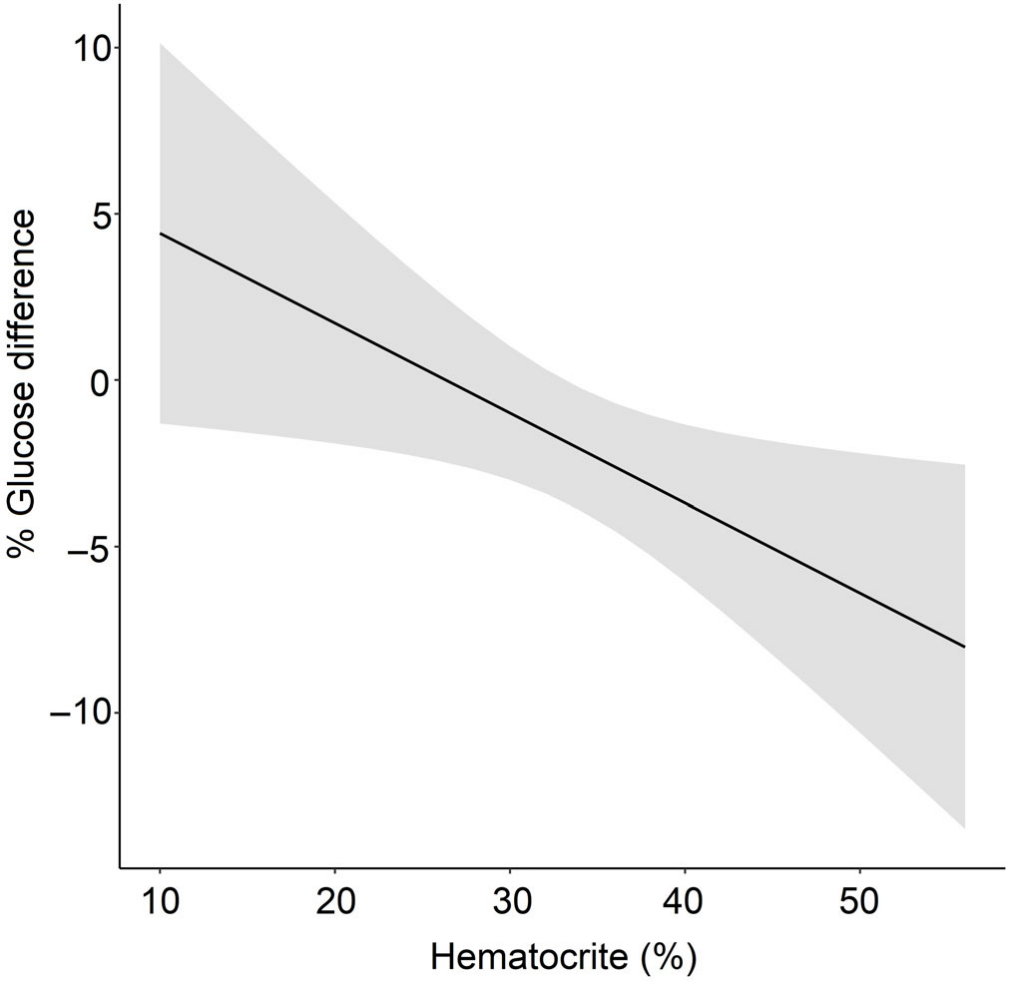
Percentage difference (%) between the point of care and reference analyzer ((GluPOC‐GluRef)/GluRef) and hematocrit value (%) in 196 calves. The line represents the significant linear association (Table 2) between hematocrit and glycemia percent difference and its associated SE shaded in gray. GluPOC, glycemia as evaluated by the point of care analyzer; GluRef, glycemia as evaluated by the reference analyzer

The distribution of the measurements between the Freestyle Optium Neo H and the Hct value is shown in Figure 2. The results of the final multivariable model include is indicated in Table 2. The adjusted coefficient of determination (R2) was only 0.02. The age, TP, and TPxHct did not reach statistical significance.

**TABLE 2:** Results of multivariable regression analysis characterizing the effect of the Hct on the percent difference between the point of care (POC) Freestyle Optium Neo H and the reference Cobas analyzer (DGlu (%) = (GluPOC‐GluRef)/GluRef)). (n = 196)

	Estimated value	SE	*P*
Intercept	7.117	4.049	.08
Hematocrit (%)	−0.270	0.117	.02

Plasma glucose concentration of 151 out of 196 sick calves were <100 mg/dL. In the evaluation of glucometer performance according to ISO 15197‐2013, 92% of the individual glucose results for the Freestyle Optium Neo H fell within 15 mg/dL of the reference method value when plasma glucose concentration <100 mg/dL (goal is 95%). 73% of the individual glucose results for the Freestyle Optium Neo H fell within 15% of the reference method value when plasma glucose concentration >100 mg/dL (goal is 95%). TEobs of the Freestyle Optium Neo H ranged from 6.0 to 43.9% (median, 14.2%). In the present study, the glucose measurements caused a TEobs ≤20 on 70.9% of samples for the Freestyle Optium Neo H.

## 4 DISCUSSION

In this study, the performance of the Freestyle Optium Neo H was assessed in measuring blood glucose concentrations in calves with different disorders. The use of 196 calves with different diseases in the present study allowed us to assess a wide range of glucose concentrations in which wide analytical range is very valuable in method comparison experiments. In this report, the range of plasma glucose concentrations was less than that in 1 study in adult cattle,^9^ but greater than that used in 2 other studies in which POC glucometers' performance were evaluated in adult cattle.^8^, ^25^

The Passing‐Bablok or Deming regression analysis that measures the systematic and proportional differences between the 2 methods is used in method comparison studies. Passing‐Bablok regression analysis revealed the constant and proportional errors in the studies evaluating agreement of different POC glucometers designed for human in dairy cows.^24^, ^26^ Deming regression for the Precision Xtra against the reference method indicated proportional error (1.11) and constant error (−17 mg/dL) in 102 periparturient dairy cows.^9^ Similarly, the Freestyle Optium Neo H yielded the negative constant and the positive proportional errors in our study and the deviation from the linearity was not significant according to Cusum test linearity (Figure 1). Our results indicated that the Freestyle Optium Neo H produced less the constant bias than that yielded by the Precision Xtra, and One Touch Vita (One Touch Vita; Lifescan, High Wycombe, UK) in the 2 previous method comparison studies.^5^, ^9^ FreeStyle Precision (Abbott GmbH & Co KG, Wiesbaden, Germany) had higher proportional (0.73) and constant (29.3 mg/dL) errors in the measurement of glucose concentration of the capillary blood in dairy cows than those produced by the Freestyle Optium Neo H in our study.^26^ Contrary to our result, it was shown that the Precision Xtra did not produce proportional bias in the measurement of blood glucose concentrations of 181 lactating dairy cows.^25^ The Precision Xtra and the reference method used glucose oxidase enzyme methodology in that study whereas in our study the enzyme methodology was different between Freestyle Optium Neo H and the reference method (glucose dehydrogenase versus hexokinase). The reference glucose measurements were performed on heparinized plasma samples in the current study; however, serum samples were used to measure reference glucose concentration in that study.^25^ In addition, in the present study the range of plasma glucose concentrations measured by the reference method was wider than that determined by the reference method in that study (18‐155 versus 16‐92 mg/dL). The reasons noted above might have contributed to this difference between our study and that study conducted by Zakian et al.^25^ When we performed the Passing‐Bablok regression analysis on <92 mg/dL plasma glucose concentration values, in our study we noticed that the proportional difference was 1.07 (95% CI 1.00‐1.16) that did not differ from 1.

The Bland‐Altman approach is also widely used in method comparison studies to quantify agreement between 2 methods and to evaluate a bias the mean differences. Bland‐Altman analysis detected that One Touch Vita underestimated blood glucose concentrations in cattle.^5^ The mean bias between the Precision Xtra and the reference method was −0.9 mg/dL in lactating dairy cows.^25^ The FreeStyle Precision Neo (Abbott Diabetes Care Ltd) also underestimated blood glucose concentrations in 97 dairy cows at the different productive stages and the mean bias was −4.50 mg/dL.^24^ Likewise, the Bland‐Altman analysis revealed a negative mean bias (−0.6 mg/dL) between the Freestyle Optium Neo H and the reference method in the measurement of blood glucose concentrations in sick calves in the present study. However, the Freestyle Optium Neo H showed positive proportional bias in the measurement of glucose concentrations (Figure 1). It was shown that a POC glucometer the Accu‐Chek Advantage (Accu‐Chek Advantage, Roche Diagnostics Corp, Indianapolis, Indiana) consistently underestimated blood glucose concentrations in a neonatal intensive care unit and the mean bias between the glucometer and the reference method was −20 mg/dL with limit of agreement range of approximately 62 mg/dL.^3^ Our results indicate that the POC glucometer performed much better in calves with different disorders than the glucometer evaluated in neonatal foals with various diseases.

Possible reasons may affect the performance of POC glucometers when whole blood is used. POC glucometers may overestimate blood glucose concentrations in anemic patients whereas underestimation may occur on hemoconcentrated blood samples. A study evaluating the use of 5 POC glucometers on dogs showed that the POC glucometers' performance was affected by the Hct in which mean difference between POC glucometers and reference method increased in low Hct values.^27^ However, PCV did not affect the performance of a POC glucometer in equine emergency patients.^4^ In periparturient dairy cows, plasma glucose concentration was a significant predictor of blood glucose concentrations measured by the Precision Xtra. In that study, however, Se analysis using a spider plot showed that the percent error in blood glucose concentration measured by the glucometer was moderately dependent on the Hct.^9^ In our study, Hct affected percent difference between the Freestyle Optium Neo H and the reference analyzer in ill calves. This relative difference could potentially have a clinical effect because of the wide range of Hct observed in calves showing that the POC analyzer overestimate by 5% the glycemia in calves with Hct close to 10% and underestimate by 7% glycemia in severely hemoconcentrated calves (with Hct value close to 55%).

It is also reported that the effect of pO_2_ on glucometer's performance depends on enzyme methodology used by glucometer.^28^ High pO_2_ can result in falsely decreased blood glucose concentrations on glucometers which has glucose oxidase enzyme methodology. Whereas glucose dehydrogenase enzyme methodology is not affected by high or low pO_2_ ^28^ and consequently it was not possible that the Freestyle Optium Neo H, which utilizes glucose dehydrogenase enzyme was affected by pO_2_.

Hypoglycemia is an important problem in neonatal calves and may require urgent clinical intervention. It was determined that plasma glucose concentration of 5040 out of 10 060 hospitalized neonatal calves were <79 mg/dL in which normoglycemia was defined as plasma glucose concentration of 79 to 124 mg/dL.^12^ Hypoglycemia can also be used as a prognostic indicator and is associated high risk of nonsurvival in neonatal calves. The odds for nonsurvival decreased by a factor of 0.71 for every 18 mg/dL increase in plasma glucose concentration in 9471 hospitalized neonatal calves with normo‐ or hypoglycemia (plasma glucose concentration <124 mg/dL).^12^ In another study, median plasma glucose concentrations of 1400 critically ill neonatal calves with diarrhea for survivors and nonsurvivors were 79 and 72 mg/dL, respectively. In that study, plasma glucose concentrations <58 mg/dL were identified as a mortality predictor.^14^ A recent study showed that diagnostic cutoff point for plasma glucose concentration for nonsurvival progression of 221 diarrheic calves with no concurrent severe disorder was set at <72 mg/dL^16^ Because hypoglycemia is not easily diagnosed based on clinical signs in ill neonatal calves, diagnosis of hypoglycemia requires blood glucose measurements.^12^ Plasma glucose measurements are performed using bench‐top autoanalyzers in diagnostic laboratories, which might be inconvenient to field practitioners and those in farm settings. POC glucometers, which can diagnose hypoglycemia with high Se and Sp might be extensively used in hospital and especially in field settings in neonatal bovine medicine. Therefore, we evaluated the diagnostic performance of the Freestyle Optium Neo H using 3 plasma glucose concentrations in this study. We established the cutoff values according to the results of previous studies evaluating the risk factors for mortality in calves affected by different diseases.^13^, ^14^, ^15^, ^16^ At the plasma glucose cutoff of <40 and <58 mg/dL, the Freestyle Optium Neo H yielded high Se and Sp. At the cutoff of <72 mg/dL, the Se and Sp of the Freestyle Optium Neo H decreased to 92.2 and 86.4%, respectively (Table 1). High AUC values indicated highly accurate diagnostic test performance of the glucometer. In another study, the Se and Sp of the FreeStyle Precision Neo were 0.91 and 0.46 in hypoglycemic (cutoff of <45 mg/dL) lactating cows, respectively.^29^ These results show that the performance of the Freestyle Optium Neo H to identify hypoglycemia in sick calves is better than the performance of the FreeStyle Precision Neo in lactating cows. Good diagnostic performance of the Freestyle Optium Neo H to diagnose hypoglycemia may have resulted from a higher intraerythrocyte glucose concentration of neonatal calves than that of adult cattle. Overall, the Freestyle Optium Neo H has clinical utility to diagnose hypoglycemia in calves.

To the best of our knowledge, only 3 studies evaluating glucometers' performance in cows reported the intra‐assay CV. In 1 of these studies, triplicate measurements of each blood sample from 97 cows were used to calculate the intra‐assay CV of the FreeStyle Precision Neo and Precision Xtra.^24^ In another study, 3 blood samples with different glucose concentrations (low, 30 mg/dL; medium, 56 mg/dL; and high, 70 mg/dL) were tested 10 times to calculate the intra‐assay CV of the FreeStyle Precision.^26^ In the third study, the intra‐assay CV of the Precision Xtra was calculated from 20 consecutive analyses of blood samples with a mean low (46 mg/dL) and moderate (102 mg/dL) blood glucose concentrations.^9^ In our study, the intra‐assay CV of the Freestyle Optium Neo H was calculated from only 7 replicates of low (mean, 38 mg/dL) and moderate (mean, 116 mg/dL) blood glucose concentrations. This is a weakness of the present study. Different CVs observed, from 3 to 7.4%, in all these studies may have been result from differences in the number of samples and replicates. Besides, the use of blood samples with different glucose concentrations in these studies may have been contributed to the differences in the observations.

Moreover, we evaluated the performance of the Freestyle Optium Neo H using performance recommendations by the ISO and the American Society for Veterinary Clinical Pathology. The Freestyle Optium Neo H did not meet the performance criteria recommended by ISO 15197‐2013; however, those criteria have been established for glucometers, which is predominantly used by individuals with diabetes mellitus. Therefore, those criteria might not be suitable the glucometers that is used on bovine medicine. However, in our report the Freestyle Optium Neo H similar to Precision Xtra^9^ and FreeStyle Precision Neo^24^ assessed in dairy cows failed to meet American Society for Veterinary Clinical Pathology recommended requirements for TEobs. Moreover, the use of incorrect algorithm for bovine on the glucometers designed for humans might cause the failure of the glucometers to meet the performance criteria.^9^

In summary, our results indicated that the Freestyle Optium Neo H had constant and proportional differences against the reference method in the measurement of blood glucose concentrations. The Bland‐Altman analysis revealed negative bias (mean, −0.6 mg/dL) between the Freestyle Optium Neo H and the reference method. The Freestyle Optium Neo H failed to meet the performance requirements recommended by the International Standards Organization and the American Society for Veterinary Clinical Pathology. These results indicate that new glucometers using calf‐specific algorithms should be developed to increase the accuracy. However, ROC analysis showed that the Freestyle Optium Neo H determined glucose concentrations indicating severe hypoglycemia and poor prognosis with high Se and Sp in calves with different diseases. Therefore, the Freestyle Optium Neo H might be used by large animal clinicians in ambulatory clinics and farm settings to detect hypoglycemia in ill calves until calf‐specific algorithms are employed in POC glucometers.

## 5 CONFLICT OF INTEREST DECLARATION

Sébastien Buczinski serves as Consulting Editor for Experimental Design and Statistics for the *Journal of Veterinary Internal Medicine*. He was not involved in review of this manuscript.

## 6 OFF‐LABEL ANTIMICROBIAL DECLARATION

Authors declare no off‐label use of antimicrobials.

## 7 INSTITUTIONAL ANIMAL CARE AND USE COMMITTEE (IACUC) OR OTHER APPROVAL DECLARATION

This study was approved by the Firat University Ethics Committee on Animal Experimentation.

## 8 HUMAN ETHICS APPROVAL DECLARATION

Authors declare human ethics approval was not needed for this study.
